# Complete Genome Sequence of *Thiohalobacter* sp. Strain COW1, Isolated from Activated Sludge Treating Coke Oven Wastewater

**DOI:** 10.1128/MRA.00013-21

**Published:** 2021-02-18

**Authors:** Kentaro Miyazaki, Hikaru Suenaga, Mamoru Oshiki, Shuichi Kawano, Toshikazu Fukushima

**Affiliations:** a Bioproduction Research Institute, National Institute of Advanced Industrial Science and Technology (AIST), Tsukuba, Ibaraki, Japan; b Department of Computational Biology and Medical Sciences, The University of Tokyo, Kashiwa, Chiba, Japan; c Cellular and Molecular Biotechnology Research Institute, National Institute of Advanced Industrial Science and Technology (AIST), Koto-ku, Tokyo, Japan; d Division of Environmental Engineering, Faculty of Engineering, Hokkaido University, Sapporo, Hokkaido, Japan; e Department of Computer and Network Engineering, Graduate School of Informatics and Engineering, The University of Electro-Communications, Chofu, Tokyo, Japan; f Environment Research Laboratory, Advanced Technology Research Laboratories, Nippon Steel Corporation, Futtsu, Chiba, Japan; Georgia Institute of Technology

## Abstract

A thiocyanate-degrading bacterium, *Thiohalobacter* sp. strain COW1, was isolated from activated sludge treating coke oven wastewater, and the complete genome sequence was determined. COW1 contained a single circular chromosome (3.23 Mb; G+C content, 63.4%) in which 2,788 protein-coding genes, 39 tRNA genes, and 3 rRNA genes were identified.

## ANNOUNCEMENT

Thiocyanate (SCN^−^) is a major component of wastewater from mining and coking industries, and it is often degraded through an activated sludge process (1). We attempted to screen for thiocyanate-degrading bacteria using activated sludge as an isolation source. More specifically, thiocyanate-degrading biomass was first enriched by operating a laboratory-scale moving bed biofilm reactor. We then obtained a highly enriched (>98.4%) culture of *Thiohalobacter* sp. strain FOKN1 by serial dilution (2). More recently, we successfully isolated *Thiohalobacter* sp. strain COW1 from the same reactor by repeating single-colony isolation on a carrageenan-solidified plate; the strain’s thiocyanate-degrading activity was confirmed experimentally. In this study, we report the complete genome sequence of the thiocyanate-degrading *Thiohalobacter* sp. strain COW1.

For genome analysis, COW1 was grown in inorganic medium (2) at 30°C for 10 days, and genomic DNA was purified using the Qiagen blood and cell culture DNA kit. Long-read sequencing and short-read sequencing were performed using GridION (Oxford Nanopore Technologies [ONT]) and DNBSEQ (MGI) systems, respectively. Default parameters were used for all software unless otherwise specified. For long-read sequencing, genomic DNA (600 ng) that had been pretreated with Short Read Eliminator (Circulomics) was used to construct a library using a ligation sequencing kit (ONT). The library was then analyzed on a FLO-MIN106 R9.41revD flow cell (ONT). Base calling was conducted using Guppy v.4.0.11 to generate 89,841 reads (506 Mb) with an average length of 5,636 bases. The raw reads were filtered (Q ≥ 10; read length,  ≥1,000 bases) using NanoFilt v.2.3.0 (3). The longest read was 221,578 bases.

For short-read sequencing, the MGIEasy FS PCR-free DNA library preparation set (MGI) was used to generate paired-end libraries (∼430-bp insert). Paired-end (2 × 150-bp) sequencing was performed on a DNBSEQ-G400RS system (MGI), yielding 17.8 million paired-end reads, spanning 2.67 Gb, with an average length of 150 bp. Raw sequencing data were processed using fastp v.0.20.1 (4) to trim adapters and low-quality data (Q ≥ 30; read length, ≥ 10 bases), yielding 13.2 million paired-end reads, spanning 1.93 Gb, with an average length of 146 bp.

The long- and short-read data were assembled *de novo* using Unicycler v.0.4.8 (5) followed by polishing with Pilon v.1.23 (6), resulting in the generation of a single circular chromosome of 3,567,646 bp (G+C content, 63.4%). Automatic annotation using DFAST v.1.2.4 (7) revealed that the chromosome contained 2,788 protein-coding genes, 39 tRNA genes, and 3 rRNA genes.

Several thiocyanate-degrading halophilic bacteria have been reported (2, 8–10), among which draft genome sequences have been reported for Thiohalobacter thiocyanaticus HRh1 (11) and *Thiohalobacter* sp. strain FOKN1 (12). JSpeciesWS analysis (13) revealed that the COW1 genome showed 98.25% average nucleotide identity (ANI) with respect to the FOKN1 genome (GenBank accession number AP018052.1) (12) and 85.91% ANI with respect to the HRh1 genome (QZMU01000001.1 and QZMU01000002.1). Taking the definition of a species with a cutoff ANI value of 95% (13), FOKN1 and COW1 belong to the same species but are distinct from HRh1.

### Data availability.

The complete genome sequence of *Thiohalobacter* sp. strain COW1 is available from DDBJ/EMBL/GenBank with accession number AP024239. Raw sequencing data were deposited in the SRA database under the accession numbers DRX248466 (Nanopore) and DRX248467 (DNBSEQ) (BioProject number PRJDB10899 and BioSample number SAMD00262787).
